# Age/BMI is a strong predictor of 30-day mortality and morbidity following total hip arthroplasty

**DOI:** 10.3389/fsurg.2025.1531104

**Published:** 2025-03-17

**Authors:** Hana M. A. Fakhoury, Mohamad Nassereddine, Hani Tamim, Ziad A. Memish, Muhammad Affan Elahi, Sarah Daher, Ali H. Hajeer

**Affiliations:** ^1^ College of Medicine, Alfaisal University, Riyadh, Saudi Arabia; ^2^ Department of Orthopedics Surgery, American University of Beirut, Beirut, Lebanon; ^3^ Biostatistics Unit, Clinical Research Institute, American University of Beirut, Beirut, Lebanon; ^4^ Department of Research, King Salman Humanitarian Aid & Relief Center, Riyadh, Saudi Arabia; ^5^ Hubert Department of Global Health, Rollins School of Public Health, Emory University, Atlanta, GA, United States; ^6^ College of Medicine, King Saud bin Abdulaziz University for Health Sciences, Ministry of National Guard Health Affairs, Riyadh, Saudi Arabia; ^7^ Department of Pathology, College of Medical Sciences, RAK Medical & Health Sciences University, Ras Al Khaimah, United Arab Emirates

**Keywords:** Hajeer score, total hip arthroplasty, postoperative outcomes, preoperative risk stratification, short-term mortality

## Abstract

**Introduction:**

This retrospective cohort study investigated the “Hajeer score” (age/BMI) as a predictor of 30-day postoperative outcomes pertaining to morbidity and mortality following total hip arthroplasty.

**Methods:**

Using the National Surgical Quality Improvement Project database from 2011 to 2021, this study analyzed perioperative factors and 30-day postoperative complications in relation to age, BMI, and the Hajeer score. The complications evaluated included venous thromboembolism, pneumonia, acute myocardial infarction, readmission rates, and 30-day mortality. Patients were categorized based on their age, BMI, and Hajeer score and adjusted odds ratios (aORS) for morbidities and mortality were calculated by multivariate logistic regression.

**Results:**

A total of 321,973 patients who underwent total hip arthroplasty were included in this study. Risk of mortality and various other outcomes (including cardiac, respiratory, urinary, and central nervous system diseases, thromboembolism, sepsis, blood transfusion, and composite morbidity) increased with age. Conversely, a higher BMI was linked to a lower risk of mortality, cardiac and respiratory diseases, and blood transfusion. A higher Hajeer score (>3) was strongly associated with an increased risk of mortality [adjusted odds ratio [OR]: 20.06, 95% confidence interval [CI]: 2.81–143.08, *p* < 0.05], cardiac diseases (adjusted OR: 8.53, 95% CI: 1.19–60.96, *p* < 0.0001), respiratory diseases (adjusted OR: 1.40, 95% CI: 1.40–1.41, *p* < 0.0001), and blood transfusion (adjusted OR: 2.12, 95% CI: 1.73–2.60, *p* < 0.05).

**Conclusion:**

The Hajeer score could be a more effective predictor of short-term (30-day) postoperative outcomes than either age or BMI alone.

## Introduction

Total hip arthroplasty (THA), also known as total hip replacement, has become a critical intervention for patients suffering from advanced degenerative joint disease. It offers significant pain relief and improved mobility (1), leading to a higher quality of life. As the number of THA procedures is projected to rise in the coming years, optimizing patient selection and perioperative care becomes increasingly important (2).

However, current methods for predicting patient outcomes after THA have limitations. Body mass index (BMI) is a commonly used metric, but research on its association with THA outcomes paints a complex picture. Studies have shown both morbid obesity and being underweight can increase mortality risk after THA (3, 4). Additionally, Hung et al. found that advanced age, rather than BMI, was a more significant risk factor for complications (5). The U-shaped relationship between BMI and all-cause mortality, with the lowest risk in the 22.5–25 kg/m^2^ range (6), further highlights the limitations of BMI as a sole predictor.

To address these limitations and potentially improve patient selection for THA, we propose the Hajeer score as a novel prognostic tool. The Hajeer score is a simple ratio of age to body mass index (age/BMI). Previous studies have shown its effectiveness in predicting mortality across diverse patient populations, including patients with COVID-19 (7) and those undergoing laparoscopic cholecystectomy (8). In this study, we leverage the National Surgical Quality Improvement Program (NSQIP) database to evaluate the Hajeer score's ability to predict postoperative complications and mortality following THA. This large database allows for a comprehensive assessment of the Hajeer score's usefulness as a prognostic tool in THA patients.

## Methods

### Study design and data collection

In this retrospective cohort study, we used the American College of Surgeons National Surgical Quality Improvement Program (ACS NSQIP) database. This prospective, validated variable-based outcomes registry provides feedback to affiliated hospitals regarding 30-day risk-adjusted surgical mortality and morbidity. It comprises acronymized data from over 600 participating non-Veteran Affairs administration hospitals for patients undergoing major surgery. The data includes patient demographics, functional statuses, laboratory data, admission sources, preoperative risk factors, perioperative variables as well as 30-day postoperative outcomes. This data is collected upon admission by trained surgical clinical reviewers from the medical record, operative log, anesthesia record, and interviews with both the surgical attending and patients (8).

In this study, we identified 321,973 patients who underwent total hip arthroplasty between 2011 and 2021 using Current Procedural Terminology (CPT) codes 27130, 27134, 27137, and 27138 maintained by the American Medical Association.

The Institutional Review Board (IRB) of the American University of Beirut (AUB) waived the need for ethical approval and a consent form, as this retrospective cohort study used de-identified data previously collected as part of a quality assurance program.

### Subgroups

Continuous variables such as age, BMI, and Hajeer (age/BMI) score were converted to categorical variables to facilitate an adjusted multivariable risk assessment for each variable. Patients were stratified by age into four groups, namely group 1 (<40 years), group 2 (40– <59.9 years), group 3 (60–79.9), and group 4 (≥80 years). Patient were also divided based on BMI into three groups i.e., underweight (<25 kg/m^2^), normal weight (25–29.9 kg/m^2^), and obese (>30 kg/m^2^). The Hajeer score was calculated by dividing age (years) by the BMI (kg/m^2^) which was used to categorize patients into 4 groups: group 1: ≤1.0, group 2: 1.1–2.0, group 3: 2.1–3.0, and group 4: >3.0. The stratification of the Hajeer score into those 4 distinctive groups was done in accordance with our previously published study about the use of the Hajeer score in patients undergoing laparoscopic cholecystectomy (8).

### Extracted variables

The collected demographic data included age, sex, height, weight, BMI, and race. The assessed characteristics included obesity, smoking status, diabetes, hypertension, steroid use, systemic sepsis within last 48 h prior surgery, dyspnea, congestive heart failure within 30 days prior surgery, severe chronic obstructive pulmonary disease (COPD), ascites, esophageal varices within last 6 months, renal failure, dialysis, impaired sensorium within last 48 h, hemiplegia, paraplegia, quadriplegia, coma lasting greater than 24 h, history of transient ischemic attacks, history of cerebrovascular accidents with neurologic deficits, tumors involving the CNS, bleeding disease, weight loss >10% within the last 6 months, disseminated cancer, chemotherapy within last 30 days, radiotherapy within the last 90 days, open wound (with or without infection), preoperative levels of serum sodium, blood urea nitrogen (BUN), total bilirubin, serum creatinine, SGOT, alkaline phosphatase, and counts of WBCs and platelets as well as INR. The operative characteristics included total operating time, inpatient/outpatient status and the American Society of Anesthesiologists (ASA) classification. The ASA score, ranging from I to V, assesses patient's overall health and comorbidities. ASA I is a healthy patient with no comorbidities, ASA II is mild systemic disease without functional limitations, ASA III is severe systemic disease with substantive functional limitations, ASA IV is severe systemic disease that is a constant threat to life, ASA V is a moribund patient not expected to survive without the operation, and ASA VI is a declared brain-dead patient whose organs are being removed for donor purposes.

The assessed outcomes in this study were: mortality, wound outcome, cardiac disease, respiratory disease, urinary disease, central nervous system (CNS) disease, thromboembolism, sepsis, and return to the operation room (OR).

### Statistical analysis

Statistical analysis was performed using Statistical Analysis Software (SAS) version 9.4. Descriptive statistics were employed, reporting number and percentages for categorical variables and means with standard deviations for continuous variables. Assessment of associations between exposures (e.g., age, BMI, Hajeer score) and the various characteristics and outcomes were assessed using the chi-square test for categorical variables, independent samples t-test for continuous variables associated with a 2-group categorical variable, or ANOVA for continuous variables associated with a group of 3 or more categorical variables.

To mitigate the confounding effects of patient characteristics, univariate and multivariate logistic regression was used given the large sample size with statistically significant or clinically relevant variables being included in the model. Adjusted odds ratios (aORS) for morbidities and mortality were calculated by logistic regression and reported with 95% confidence intervals (CIs) with the level of significance for the *p*-value being set at <0.05. The odds ratios were adjusted for the following variables: congestive heart failure within 30 days prior to surgery, severe COPD, preoperative acute renal failure, bleeding disorders, cancer, ascites within 30 days before surgery, as well as preoperative treatments (e.g., dialysis, ventilation, chemotherapy, and radiation therapy), and the aforementioned ASA classification. For the multivariate analysis, missing data were replaced by the average for continuous variable, or the null value for categorical variables. It is worth nothing, that most of the variables included in the model had very little missing data (<1%).

## Results

321,973 patients who underwent total hip arthroplasty were included in this study. The baseline demographics are presented in Table 1 and stratified by the Hajeer score. As the Hajeer score increased, the surgical duration, smoking prevalence, and COPD decreased, while the prevalence of hypertension and transfusions increased across the groups.

**Table 1:** Baseline characteristics of patients stratified by Hajeer score.

Hajeer score groups						
Variables	Total	Group 1: ≤1.0	Group 2: 1.1–2.0	Group 3: 2.1–3.0	Group 4: >3.0	*P* value
Number	321,973	3,308	116,608	157,496	44,561	
Age, mean (SD)	65.45 (11.57)	35.43 (10.79)	57.33 (9.34)	68.49 (8.19)	78.19 (7.63)	<0.0001
Sex (female), *n* (%)	176,598 (54.86)	1,704 (51.53)	56,084 (48.11)	86,806 (55.13)	32,004 (71.86)	<0.0001
Height (inch), mean (SD)	66.13 (4.15)	65.96 (4.87)	66.72 (4.20)	66.10 (4.08)	64.75 (3.86)	<0.0001
Weight (lb), mean (SD)	188.7 (46.16)	256.03 (75.56)	222.91 (41.85)	176.60 (30.24)	136.94 (23.65)	<0.0001
BMI, mean (SD)	30.2 (6.34)	41.36 (11.75)	35.18 (5.71)	28.35 (3.67)	22.87 (2.75)	<0.0001
Hajeer score, mean (SD)	2.27 (0.67)	0.86 (0.13)	1.65 (0.24)	2.43 (0.28)	3.45 (0.42)	<0.0001
Work relative value unit, mean (SD)	21.47 (2.19)	21.59 (2.39)	21.42 (2.11)	21.44 (2.15)	21.68 (2.49)	<0.0001
Surgical duration (min), mean (SD)	96.43 (45.97)	122.43 (62.22)	102.59 (47.60)	92.80 (43.65)	91.19 (45.73)	<0.0001
Race (white), *n* (%)	237,037 (73.62)	2,155 (65.15)	84,084 (72.11)	116,813 (74.17)	33,985 (76.27)	<0.0001
Smoking, *n* (%)	40,888 (12.7)	835 (25.24)	19,243 (16.50)	17,352 (11.02)	3,458 (7.76)	<0.0001
Diabetes, *n* (%)	39,469 (12.26)	298 (9.01)	17,953 (15.40)	17,870 (11.35)	3,348 (7.51)	<0.0001
Hypertension, *n* (%)	179,393 (55.72)	1,048 (31.68)	64,532 (55.34)	88,034 (55.90)	25,779 (57.85)	<0.0001
Systemic sepsis within the last 48 h, *n* (%)	2,358 (0.73)	27 (0.82)	664 (0.57)	1,028 (0.65)	639 (1.43)	<0.0001
Steroid use, *n* (%)	12,568 (3.9)	295 (8.92)	4,411 (3.78)	5,872 (3.73)	1,990 (4.47)	<0.0001
Operation within the last 30 days, *n* (%)	305,527 (94.89)	3,107 (93.92)	110,902 (95.11)	149,483 (94.91)	42,035 (94.33)	<0.0001
Functional status (totally dependent), *n* (%)	511 (0.16)	4 (0.12)	85 (0.07)	199 (0.13)	223 (0.50)	<0.0001
ASA IV/V, *n* (%)	7,534 (2.34)	108 (3.27)	2,199 (1.89)	3,293 (2.09)	1,934 (4.34)	<0.0001
Anesthesia, *n* (%)	159,824 (49.64)	2,097 (63.39)	61,355 (52.62)	75,016 (47.63)	21,356 (47.93)	<0.0001
Inpatient, *n* (%)	303,293 (94.2)	3,109 (93.98)	109,417 (93.83)	148,227 (94.11)	42,540 (95.46)	<0.0001
Days from hospital admission to surgery (>1), *n* (%)	7,233 (2.25)	68 (2.06)	1,502 (1.29)	3,259 (2.07)	2,404 (5.40)	<0.0001
Airway trauma, *n* (%)	239,513 (98.39)	2,371 (97.77)	86,667 (98.39)	118,003 (98.47)	32,472 (98.13)	<0.0001
Transfusion, *n* (%)	919 (0.29)	21 (0.63)	215 (0.18)	355 (0.23)	328 (0.74)	<0.0001
Wound infection, *n* (%)	1,991 (0.62)	43 (1.30)	792 (0.68)	840 (0.53)	316 (0.71)	<0.0001
Dyspnea, *n* (%)	15,070 (4.68)	168 (5.08)	5,709 (4.90)	6,927 (4.40)	2,266 (5.09)	<0.0001
Congestive heart failure within the last 30 days, *n* (%)	1,383 (0.43)	9 (0.27)	365 (0.31)	631 (0.40)	378 (0.85)	<0.0001
Angina within the last 30 days, *n* (%)	305,679 (94.94)	3,108 (93.95)	110,949 (95.15)	149,580 (94.97)	42,042 (94.35)	<0.0001
Myocardial infarction within the last 6 months, *n* (%)	305,637 (94.93)	3,108 (93.95)	110,940 (95.14)	149,551 (94.96)	42,038 (94.34)	<0.0001
Previous percutaneous coronary intervention, *n* (%)	306,570 (95.22)	3,110 (94.01)	111,169 (95.34)	150,086 (95.30)	42,205 (94.71)	<0.0001
Previous cardiac surgery, *n* (%)	306,378 (95.16)	3,111 (94.04)	111,085 (95.26)	149,973 (95.22)	42,209 (94.72)	<0.0001
History of peripheral vascular disease, *n* (%)	305,741 (94.96)	3,108 (93.95)	110,958 (95.15)	149,609 (94.99)	42,066 (94.40)	<0.0001
Rest pain or gangrene, *n* (%)	305,614 (94.92)	3,108 (93.95)	110,935 (95.13)	149,539 (94.95)	42,032 (94.32)	<0.0001
Current pneumonia, *n* (%)	305,621 (94.92)	3,109 (93.98)	110,935 (95.13)	149,544 (94.95)	42,033 (94.33)	<0.0001
COPD, *n* (%)	13,139 (4.08)	50 (1.51)	3,974 (3.41)	6,570 (4.17)	2,545 (5.71)	<0.0001
Ventilator-dependent within the last 48 h, *n* (%)	42 (0.01)	1 (0.03)	12 (0.01)	22 (0.01)	7 (0.02)	0.6209
Ascites, *n* (%)	89 (0.03)	0 (0.00)	21 (0.02)	49 (0.03)	19 (0.04)	0.0275
Esophageal varices within the last 6 months, *n* (%)	305,616 (94.92)	3,108 (93.95)	110,936 (95.14)	149,541 (94.95)	42,031 (94.32)	<0.0001
Renal failure, *n* (%)	214 (0.07)	3 (0.09)	57 (0.05)	96 (0.06)	58 (0.13)	<0.0001
Currently on dialysis, *n* (%)	918 (0.29)	23 (0.70)	307 (0.26)	433 (0.27)	155 (0.35)	<0.0001
Impaired sensorium within the last 48 h, *n* (%)	305,630 (94.92)	3,108 (93.95)	110,934 (95.13)	149,549 (94.95)	42,039 (94.34)	<0.0001
Hemiplegia, *n* (%)	305,680 (94.94)	3,108 (93.95)	110,951 (95.15)	149,578 (94.97)	42,043 (94.35)	<0.0001
Paraplegia, *n* (%)	305,656 (94.93)	3,111 (94.04)	110,949 (95.15)	149,558 (94.96)	42,038 (94.34)	<0.0001
Quadriplegia, *n* (%)	305,612 (94.92)	3,108 (93.95)	110,933 (95.13)	149,539 (94.95)	42,032 (94.32)	<0.0001
Coma lasting >24 h, *n* (%)	305,611 (94.92)	3,108 (93.95)	110,933 (95.13)	149,539 (94.95)	42,031 (94.32)	<0.0001
History of transient ischemic attacks, *n* (%)	306,040 (95.05)	3,110 (94.01)	111,009 (95.20)	149,765 (95.09)	42,156 (94.60)	<0.0001
History of CVA with neurological deficit, *n* (%)	305,812 (94.98)	3,108 (93.95)	110,971 (95.17)	149,642 (95.01)	42,091 (94.46)	<0.0001
History of CVA without neurological deficit, *n* (%)	305,928 (95.02)	3,109 (93.98)	110,992 (95.18)	149,713 (95.06)	42,114 (94.51)	<0.0001
Tumor involving the CNS, *n* (%)	305,622 (94.92)	3,108 (93.95)	110,938 (95.14)	149,543 (94.95)	42,033 (94.33)	<0.0001
Bleeding disease, *n* (%)	7,823 (2.43)	53 (1.60)	2,128 (1.82)	3,924 (2.49)	1,718 (3.86)	<0.0001
Weight loss >10% within the last 6 months, *n* (%)	890 (0.28)	5 (0.15)	163 (0.14)	329 (0.21)	393 (0.88)	<0.0001
Disseminated cancer, *n* (%)	1,423 (0.44)	41 (1.24)	374 (0.32)	748 (0.47)	260 (0.58)	<0.0001
Chemotherapy within the last 30 days, *n* (%)	305,659 (94.93)	3,109 (93.98)	110,943 (95.14)	149,565 (94.96)	42,042 (94.35)	<0.0001
Radiotherapy within the last 90 days, *n* (%)	305,819 (94.98)	3,110 (94.01)	110,992 (95.18)	149,638 (95.01)	42,079 (94.43)	<0.0001
Open wound (with or without infection), *n* (%)	1,682 (0.52)	25 (0.76)	513 (0.44)	709 (0.45)	435 (0.98)	<0.0001
Preoperative serum sodium level (≤135 mmol/L), *n* (%)	24,012 (8.04)	184 (6.22)	6,090 (5.66)	11,608 (7.94)	6,130 (14.65)	<0.0001
Preoperative serum sodium level (>145 mmol/L), *n* (%)	2,585 (0.8)	17 (0.51)	902 (0.77)	1,327 (0.84)	339 (0.76)	0.0315
Preoperative BUN level (>40 mmol/L), *n* (%)	3,357 (1.2)	23 (0.81)	888 (0.87)	1,625 (1.19)	821 (2.10)	<0.0001
Preoperative serum creatinine level (>1.2 mg/dl), *n* (%)	28,476 (9.43)	138 (4.60)	8,908 (8.19)	14,813 (10.02)	4,617 (10.94)	<0.0001
Preoperative total bilirubin level (>1 mg/dl), *n* (%)	9,491 (6.25)	98 (6.22)	3,230 (5.77)	4,763 (6.52)	1,400 (6.61)	<0.0001
Preoperative SGOT level (>40 U/L), *n* (%)	9,206 (5.97)	173 (10.83)	4,163 (7.31)	3,829 (5.16)	1,041 (4.84)	<0.0001
Preoperative alkaline phosphatase level (>125 U/L), *n* (%)	10,221 (6.7)	131 (8.35)	3,882 (6.90)	4,616 (6.28)	1,592 (7.48)	<0.0001
Preoperative WBC count (>11), *n* (%)	13,660 (4.47)	288 (9.36)	5,196 (4.72)	5,818 (3.88)	2,358 (5.53)	<0.0001
Preoperative platelet count (≤150), *n* (%)	14,797 (4.84)	92 (2.99)	4,455 (4.04)	7,495 (5.00)	2,755 (6.46)	<0.0001
Preoperative international normalized ratio (>1.4), *n* (%)	4,107 (2.14)	22 (1.17)	1,123 (1.66)	2,092 (2.22)	870 (3.09)	<0.0001

Hajeer score groups are defined as follows: ≤1.0, 1.1–2.0, 2.1–3.0, and >3.0. Data are presented as mean (SD) for continuous variables and number (percentage) for categorical variables.

ASA, American Society of Anesthesiologists; COPD, chronic obstructive pulmonary disease; CVA, cerebrovascular accident; BUN, blood urea nitrogen; SGOT, serum glutamic-oxaloacetic transaminase; WBC, white blood cell.

### Outcomes by age

Table 2 summarizes the results of the ordered regression analysis of the age groups with various outcomes. As age increased, a greater prevalence of cardiac disease, respiratory disease, urinary disease, and blood transfusion was noted. This was particularly striking in the age ≥80 years group with the following adjusted odds ratios: mortality (OR 3.25, CI 2.71–2.90), cardiac disease (OR 1.88, CI 1.49–2.38), respiratory disease (OR 1.55, CI 1.29–1.88), urinary disease (OR 1.29, CI 0.91–1.82), and blood transfusion (OR 1.57, CI 1.48–1.67). On the other hand, older patients were less likely to develop adverse wound outcomes when compared to younger patients.

**Table 2:** Effect of Age on postoperative outcomes following total Hip arthroplasty.

Age groups				
Outcome	<40 years	40–59.9 years	60–79.9 years	≥80 years
Mortality, *n* (%)	3 (0.04)	53 (0.06)	290 (0.15)	342 (0.95)
Unadjusted OR	Reference	0.73 (0.53–1.02)	2.29 (1.84–2.85)*	4.36 (3.65–5.21)*
Adjusted OR	Reference	0.81 (0.58–1.13)	1.86 (1.49–2.31)*	3.25 (2.71–3.90)*
Wound outcome, *n* (%)	86 (1.24)	794 (0.92)	1,567 (0.81)	286 (0.80)
Unadjusted OR	Reference	1.05 (0.95–1.16)	0.90 (0.83–0.97)**	1.03 (0.90–1.18)
Adjusted OR	Reference	1.09 (0.93–1.28)	0.81 (0.71–0.92)**	0.64 (0.51–0.80)**
Cardiac disease, *n* (%)	5 (0.07)	97 (0.11)	585 (0.30)	414 (1.15)
Unadjusted OR	Reference	0.64 (0.50–0.81)**	2.16 (1.85–2.52)*	2.68 (2.33–3.09)*
Adj OR	Reference	0.60 (0.41–0.89)**	1.68 (1.32–2.15)*	1.88 (1.49–2.38)*
Respiratory disease, *n* (%)	35 (0.50)	234 (0.27)	901 (0.47)	469 (1.31)
Unadjusted	Reference	0.75 (0.64–0.88)**	1.49 (1.33–1.67)*	2.26 (2.00–2.56)*
Adjusted	Reference	0.74 (0.58–0.95)**	1.17 (0.99–1.39)	1.55 (1.29–1.88)*
Urinary disease, *n* (%)	7 (0.10)	105 (0.12)	293 (0.15)	126 (0.35)
Unadjusted OR	Reference	0.95 (0.74–1.22)	1.27 (1.05–1.54)**	2.04 (1.62–2.58)*
Adjusted OR	Reference	0.94 (0.64–1.38)	0.92 (0.70–1.22)	1.29 (0.91–1.82)
CNS disease, *n* (%)	3 (0.04)	43 (0.05)	204 (0.11)	118 (0.33)
Unadjusted OR	Reference	0.81 (0.56–1.19)	2.18 (1.69–2.82)*	2.16 (1.69–2.77)*
Adjusted OR	Reference	1.04 (0.55–1.95)	2.10 (1.33–3.32)**	2.13 (1.41–3.24)**
Thromboembolism, *n* (%)	30 (0.43)	388 (0.45)	1,203 (0.62)	365 (1.02)
Unadjusted OR	Reference	0.89 (0.78–1.02)	1.37 (1.24–1.50)*	1.39 (1.22–1.58)*
Adjusted OR	Reference	0.91 (0.74–1.12)	1.37 (1.17–1.59)*	1.05 (0.86–1.28)
Sepsis, *n* (%)	53 (0.76)	318 (0.37)	773 (0.40)	283 (0.79)
Unadjusted OR	Reference	0.93 (0.81–1.08)	1.06 (0.95–1.19)	1.85 (1.59–2.15)*
Adjusted OR	Reference	0.93 (0.73–1.17)	0.90 (0.75–1.07)	1.27 (1.00–1.60)**
Blood transfusion, *n* (%)	687 (9.91)	5,302 (6.17)	13,596 (7.03)	5,391 (15.02)
Unadjusted OR	Reference	0.93 (0.89–0.96)*	1.13 (1.10–1.17)*	2.14 (2.06–2.22)*
Adjusted OR	Reference	0.87 (0.82–0.92)*	1.13 (1.08–1.18)*	1.57 (1.48–1.67)*
Return to OR, *n* (%)	164 (2.37)	1,806 (2.10)	4,186 (2.17)	1,081 (3.01)
Unadjusted OR	Reference	1.01 (0.95–1.08)	1.07 (1.02–1.13)**	1.34 (1.25–1.44)*
Adjusted OR	Reference	1.00 (0.90–1.11)	1.01 (0.93–1.09)	0.98 (0.87–1.10)
Composite morbidity, *n* (%)	180 (2.60)	1,668 (1.94)	4,632 (2.40)	1,650 (4.60)
Unadjusted OR	Reference	0.91 (0.85–0.97)**	1.21 (1.16–1.27)*	1.75 (1.64–1.86)*
Adjusted OR	Reference	0.93 (0.84–1.03)	1.08 (1.00–1.17)**	1.18 (1.07–1.31)**

ORs are shown for the 40–59.9 years, 60–79.9 years, and ≥80 years groups compared to the reference group (<40 years old). Both adjusted and unadjusted ORs are included.

The variables considered and adjusted in the multivariate analysis were underlying medical conditions (congestive heart failure within 30 days prior to surgery, severe COPD, preoperative acute renal failure, bleeding disorders, cancer, and ascites within 30 days prior to surgery), preoperative treatments (dialysis, ventilation, chemotherapy, and radiation therapy), and ASA classification.

* *p* < 0.0001, **p < 0.05.

### Outcomes by BMI

Table 3 presents the postoperative outcomes following total hip arthroplasty categorized by BMI. While the risk of wound infection, urinary disease, return to the OR and composite morbidity significantly increased as the BMI increased, however, a decline in mortality, blood transfusion, and cardiac disease was observed as the BMI increased. The adjusted odds ratios (OR) for BMI > 30 group were as follows: mortality (OR 0.39, CI 0.34–0.45), wound outcome (OR 2.13, CI 1.79–2.53), cardiac disease (OR 0.62 CI 0.51–0.76), respiratory disease (OR 0.70, CI 0.60–0.81), urinary disease (OR 1.67, CI 1.19–2.35), CNS disease (OR 0.62, CI 0.43–0.89), blood transfusion (OR 0.51, CI 0.49–0.54), return to OR (OR 1.18, CI 1.08–1.29), and composite morbidity (OR 1.16, CI 1.07–1.27).

**Table 3:** Effect of BMI on postoperative outcomes following total Hip arthroplasty.

BMI groups			
Outcome	<25 kg/m^2^	25–29.9 kg/m^2^	>30 kg/m^2^
Mortality, *n* (%)	270 (0.41)	196 (0.18)	222 (0.15)
Unadjusted OR	Reference	1.25 (1.03–1.51)**	0.38 (0.33–0.44)*
Adjusted OR	Reference	1.47 (1.22–1.78)*	0.39 (0.34–0.45)*
Wound outcome, *n* (%)	360 (0.54)	613 (0.57)	1,760 (1.18)
Unadjusted OR	Reference	0.49 (0.45–0.54)*	2.01 (1.80–2.24)*
Adjusted OR	Reference	0.52 (0.44–0.61)*	2.13 (1.79–2.53)*
Cardiac disease, *n* (%)	288 (0.44)	362 (0.34)	451 (0.30)
Unadjusted OR	Reference	1.12 (0.97–1.28)	0.72 (0.63–0.82)*
Adjusted OR	Reference	1.32 (1.05–1.66)**	0.62 (0.51–0.76)*
Respiratory disease, *n* (%)	454 (0.69)	495 (0.46)	690 (0.46)
Unadjusted OR	Reference	1.01 (0.90–1.13)	0.68 (0.61–0.75)*
Adjusted OR	Reference	1.18 (0.99–1.40)	0.70 (0.60–0.81)*
Urinary disease, *n* (%)	76 (0.11)	138 (0.13)	317 (0.21)
Unadjusted OR	Reference	0.62 (0.51–0.75)*	1.68 (1.33–2.13)*
Adjusted OR	Reference	0.82 (0.61–1.09)	1.67 (1.19–2.35)**
CNS disease, *n* (%)	83 (0.13)	117 (0.11)	168 (0.11)
Unadjusted OR	Reference	1.00 (0.79–1.27)	0.84 (0.66–1.07)
Adjusted OR	Reference	1.35 (0.90–2.02)	0.62 (0.43–0.89)**
Thromboembolism, *n* (%)	350 (0.53)	637 (0.60)	999 (0.67)
Unadjusted OR	Reference	0.90 (0.81–0.99)**	1.23 (1.09–1.37)**
Adjusted OR	Reference	0.98 (0.84–1.15)	1.32 (1.11–1.58)**
Sepsis, *n* (%)	314 (0.47)	368 (0.35)	745 (0.50)
Unadjusted OR	Reference	0.71 (0.62–0.80)*	0.98 (0.86–1.10)
Adjusted OR	Reference	0.80 (0.66–0.97)**	1.04 (0.87–1.24)
Blood transfusion, *n* (%)	7,596 (11.49)	7,913 (7.42)	9,467 (6.35)
Unadjusted OR	Reference	1.18 (1.15–1.22)*	0.54 (0.53–0.56)*
Adjusted OR	Reference	1.34 (1.27–1.40)*	0.51 (0.49–0.54)*
Return to OR, *n* (%)	1,375 (2.08)	2,002 (1.88)	3,860 (2.59)
Unadjusted OR	Reference	0.73 (0.69–0.77)*	1.20 (1.13–1.27)*
Adjusted OR	Reference	0.76 (0.70–0.84)*	1.18 (1.08–1.29)**
Composite morbidity, *n* (%)	1,576 (2.38)	2,292 (2.15)	4,262 (2.86)
Unadjusted OR	Reference	0.76 (0.72–0.79)*	1.15 (1.09–1.22)*
Adjusted OR	Reference	0.86 (0.79–0.94)**	1.16 (1.07–1.27)**

ORs are shown for the normal weight (25–29.9 kg/m^2^) and obese (>30 kg/m^2^) groups compared to the underweight reference group (<25 kg/m^2^). Both adjusted and unadjusted ORs are included.

The variables considered and adjusted in the multivariate analysis were underlying medical conditions (congestive heart failure within 30 days prior to surgery, severe COPD, preoperative acute renal failure, bleeding disorders, cancer, and ascites within 30 days prior to surgery), preoperative treatments (dialysis, ventilation, chemotherapy, and radiation therapy), and ASA classification.

*p < 0.0001, **p < 0.05.

### Outcomes by Hajeer score

Table 4 outlines the results of the ordered regression analysis of Hajeer score groups with different outcomes. As the Hajeer score increased, there was a greater risk of respiratory disease, cardiac disease, and composite morbidity decreased. Conversely, a decrease in the risk of wound outcomes was associated with an increasing Hajeer score. However, the risk of composite morbidity, CNS diseases, return to OR, sepsis, thromboembolism, and urinary issues did not significantly vary across the Hajeer score groups. The adjusted odds rations for Hajeer score group 4 were as follows: mortality (OR 20.06, CI 2.81–143.08), wound outcome (OR 0.23, CI 0.15–0.36), cardiac disease (OR 8.53, CI 1.19–60.96), respiratory disease (OR 1.40, CI 1.40–1.41), and blood transfusion (OR 2.12, CI 1.73–2.60).

**Table 4:** Association between hajeer score and postoperative outcomes following total Hip arthroplasty.

Hajeer score groups				
Outcome	Group 1: ≤1.0	Group 2: 1.1–2.0	Group 3: 2.1–3.0	Group 4: >3.0
Mortality, *n* (%)	1 (0.03)	108 (0.09)	263 (0.17)	316 (0.71)
Unadjusted OR	Reference	3.06 (0.43–21.86)	5.52 (0.78–39.23)	23.56 (3.32–167.38)**
Adjusted OR	Reference	3.56 (0.50–25.53)	6.38 (0.89–45.53)	20.06 (2.81–143.08)**
Wound outcome, *n* (%)	69 (2.09)	1,365 (1.17)	1,050 (0.67)	249 (0.56)
Unadjusted OR	Reference	0.56 (0.44–0.71)*	0.32 (0.25–0.40)*	0.26 (0.20–0.35)*
Adjusted OR	Reference	0.68 (0.46–1.03)	0.36 (0.24–0.55)*	0.23 (0.15–0.36)*
Cardiac disease, *n* (%)	2 (0.06)	218 (0.19)	524 (0.33)	357 (0.80)
Unadjusted OR	Reference	3.09 (0.77–12.44)	5.51 (1.38–22.10)**	13.34 (3.32–53.53)**
Adjusted OR	Reference	2.53 (0.35–18.14)	4.19 (0.59–29.89)	8.53 (1.19–60.96)**
Respiratory disease, *n* (%)	12 (0.36)	415 (0.36)	763 (0.48)	449 (1.01)
Unadjusted OR	Reference	0.98 (0.55–1.74)	1.34 (0.76–2.37)	2.80 (1.57–4.97)**
Adjusted OR	Reference	0.85 (0.85–0.85)**	0.93 (0.93–0.94)**	1.40 (1.40–1.41)**
Urinary disease, *n* (%)	12 (0.36)	198 (0.17)	232 (0.15)	89 (0.20)
Unadjusted OR	Reference	0.47 (0.26–0.84)**	0.41 (0.23–0.73)**	0.55 (0.30–1.01)
Adjusted OR	Reference	0.53 (0.21–1.31)	0.41 (0.16–1.01)	0.41 (0.16–1.04)
CNS disease, *n* (%)	1 (0.03)	80 (0.07)	182 (0.12)	105 (0.24)
Unadjusted OR	Reference	2.27 (0.32–16.32)	3.83 (0.54–27.31)	7.81 (1.09–55.98)**
Adjusted OR	Reference	0.96 (0.13–7.08)	1.43 (0.20–10.35)	3.29 (0.45–23.97)
Thromboembolism, *n* (%)	16 (0.48)	638 (0.55)	989 (0.63)	343 (0.77)
Unadjusted OR	Reference	1.13 (0.69–1.86)	1.30 (0.79–2.13)	1.60 (0.97–2.64)
Adjusted OR	Reference	1.22 (0.57–2.60)	1.45 (0.69–3.08)	1.48 (0.69–3.18)
Sepsis, *n* (%)	33 (1.00)	541 (0.46)	567 (0.36)	286 (0.64)
Unadjusted OR	Reference	0.46 (0.33–0.66)*	0.36 (0.25–0.51)*	0.64 (0.45–0.92)**
Adjusted OR	Reference	0.81 (0.43–1.56)	0.61 (0.32–1.17)	0.74 (0.38–1.44)
Blood transfusion, *n* (%)	294 (8.89)	6,896 (5.91)	11,457 (7.27)	6,329 (14.20)
Unadjusted OR	Reference	0.64 (0.57–0.73)*	0.80 (0.71–0.91)**	1.70 (1.50–1.92)*
Adjusted OR	Reference	0.83 (0.68–1.01)	1.17 (0.96–1.43)	2.12 (1.73–2.60)*
Return to OR, *n* (%)	116 (3.51)	2,879 (2.47)	3,135 (1.99)	1,107 (2.48)
Unadjusted OR	Reference	0.70 (0.58–0.84)**	0.56 (0.46–0.68)*	0.70 (0.58–0.85)**
Adjusted OR	Reference	0.90 (0.65–1.25)	0.73 (0.53–1.02)	0.73 (0.52–1.02)
Composite morbidity, *n* (%)	118 (3.57)	2,891 (2.48)	3,615 (2.30)	1,506 (3.38)
Unadjusted OR	Reference	0.69 (0.57–0.83)*	0.64 (0.53–0.77)*	0.95 (0.78–1.14)
Adjusted OR	Reference	0.85 (0.62–1.17)	0.78 (0.57–1.07)	0.87 (0.63–1.21)

ORs are presented for Hajeer score groups 2 (1.1–2.0), 3 (2.1–3.0), and 4 (>3.0) compared to the reference group (Hajeer score ≤1.0). Both adjusted and unadjusted ORs are included.

The variables considered and adjusted in the multivariate analysis were underlying medical conditions (congestive heart failure within 30 days prior to surgery, severe COPD, preoperative acute renal failure, bleeding disorders, cancer, and ascites within 30 days prior to surgery), preoperative treatments (dialysis, ventilation, chemotherapy, and radiation therapy), and ASA classification.

* *p* < 0.0001, **p < 0.05.

## Discussion

Total hip arthroplasty (THA) is one of the most successful orthopedic procedures and involves resection of disease articular surfaces of the hip with subsequent replacement with prosthetic hip components. In a population-based cohort study with over 60,000 patients who underwent THA, over 90% of patients had successful results, were pain-free, and developed no complications within a 15-year postoperative period (9).

This study investigated the association between age, BMI, and the Hajeer score with postoperative outcomes following THA. Our results demonstrate that a higher Hajeer score is a strong predictor of increased risk cardiac and respiratory complications, as well as blood transfusions. These findings suggest that the Hajeer score, incorporating both age and BMI, offers a more comprehensive assessment of patient risk compared to either factor alone.

Increasing age was associated with a broader spectrum of negative outcomes, including mortality, various organ system complications, thromboembolism, sepsis, and blood transfusion. Conversely, a higher BMI was associated with a decreased risk of mortality and cardiac and respiratory complications.

Our findings align with several previously published studies demonstrating that overweight and obese patients have a higher long-term survival rate (10, 11), including after surgery (12). Conversely, those who are underweight face a higher risk of mortality (10) and developing post-operative complications (12) when compared to individuals with a higher BMI. A 2022 meta-analysis by Yang et al. reported an inverse association between body weight and postoperative mortality in older adults undergoing hip fracture surgery (13). Similarly, Woo et al. (4) found that underweight (BMI < 18.5 kg/m^2^) was a significant risk factor for mortality after THA in older individuals (4).

A study conducted in Australia on patients undergoing primary THA found that those who were classified as overweight or obese class II had lower odds of perioperative complications, especially cardiac complications (14). Similarly, an earlier study also using data from the ACS-NSQIP database by Scully et al. (15) analyzed the impact of BMI on 30-day complications in THA patients, albeit with a smaller sample size and shorter duration (15). Their findings revealed a nonlinear J-shaped relationship between BMI and complications such as readmission, reoperation, superficial infection, prosthetic joint infection, and sepsis, with the lowest risk observed in patients with a BMI of 27–28 kg/m^2^ (15). They also found that BMI was a more effective predictor when treated as a continuous variable rather than a categorical variable, indicating that the risk of mortality and blood transfusion had a reverse J-shaped relationship with BMI (15). Moreover, those studies have also highlighted the limitations of using BMI alone as a predictor, especially in older individuals, emphasizing the need for a more nuanced approach to risk assessment in this population (10–12).

To further clarify these associations, it is important to explore the underlying biological mechanisms driving these outcomes. The “obesity paradox” concept has been illustrated in multiple studies and may be applicable in this context as well. Obesity is thought to confer a paradoxical protective effect in certain populations by providing greater metabolic reserves during physiological stress, as evidenced by studies from Yang et al. (13) and Modig et al. (16). The latter study specifically examined patients with hip fractures and found that overweight and even obese patients had better one-year survival rates compared to those with a normal BMI, whereas a low BMI was associated with worse survival outcomes (16). Additionally, patients with a BMI below 22 kg/m^2^ had a reduced likelihood of returning home after a fracture (16).

The “obesity paradox” may also be explained by the role of chronic low-grade inflammation commonly observed in individuals with higher BMI. This baseline inflammatory state may prime the immune system to respond more efficiently to surgical stress, ultimately leading to a protective effect (8). Furthermore, lower levels of natriuretic peptides in obese individuals may provide additional protection against endotoxin- and cytokine-mediated inflammation (13), further supporting the paradoxical benefits of obesity in certain clinical scenarios.

Nonetheless, the paradoxical association between BMI and mortality highlights potential risks at both ends of the spectrum. Woo et al. (4) also reported increased short-term mortality risk for obese patients (BMI > 30 kg/m^2^) (4). On the other hand, underweight individuals undergoing total knee arthroplasty are at higher risk of surgical site infections and blood transfusions, possibly due to lower preoperative hemoglobin levels (17).

The association between lower body weight and poorer outcomes in older adults is likely due to several factors. Age-related muscle loss (sarcopenia) contributes to decreased functional capacity and may hinder recovery from surgery (18, 19). In individuals with already low body weight, this muscle loss can further increase mortality risk (20). Frailty, characterized by sarcopenia, is highly prevalent in older, underweight individuals and is a significant risk factor for poor surgical outcomes (10, 21, 22). Sarcopenia has been linked to delayed wound healing, increased susceptibility to infections, and prolonged recovery times (23, 24) possibly due to their low immunity as evidenced by a reduction in cellular immune function due to decreased glutamine and an increased level of proinflammatory mediators (24, 25). In addition, sarcopenic patients are at risk of postoperative pulmonary complications such as aspiration and atelectasis due in part to impaired respiratory muscle function (25). Malnutrition, often associated with low BMI, can also be an overlapping condition contributing to poorer outcomes in older individuals (11, 16, 22).

Regular physical activity, even at modest levels, can help frail older adults maintain muscle mass and mitigate the negative effects of aging and disease on body composition (26). Exercise interventions may improve tolerance to surgery and potentially reduce postoperative complications (26).

Overall, our findings are also consistent with another published study on patients undergoing laparoscopic cholecystectomy, another relatively safe surgical procedure in which an increased Hajeer score was strongly associated with an increased risk of mortality and composite morbidity (8).

Despite a large sample size (*n* = 321,973), the relatively low number of mortality and morbidity events within the NSQIP database limited our ability to detect some associations. Additionally, the database only provides information on 30-day outcomes. Future studies with longer follow-up periods (>30 days) are warranted to confirm these findings and explore the broader utility of the Hajeer score. Investigating the Hajeer score's effectiveness in predicting outcomes in other surgical procedures would be valuable.

In conclusion, the Hajeer score, combining age and BMI, emerged as a strong predictor of postoperative outcomes after THA, outperforming individual measures of age or BMI. This score has the potential to improve preoperative risk stratification. However, further research with longer follow-up periods and a broader range of surgical procedures is necessary to fully validate these findings and explore the Hajeer score's potential applications.

## Data Availability

The data for this study was obtained from the American College of Surgeons National Surgical Quality Improvement Program (ACS NSQIP) Participant Use Data File.

## References

[1] Yan L Ge L Dong S Saluja K Li D Reddy KS Evaluation of comparative efficacy and safety of surgical approaches for total hip arthroplasty: a systematic review and network meta-analysis. JAMA Netw Open. (2023) 6(1):e2253942. 10.1001/jamanetworkopen.2022.53942 36719679 PMC9890287

[2] Shichman I Roof M Askew N Nherera L Rozell JC Seyler TM Projections and epidemiology of primary hip and knee arthroplasty in medicare patients to 2040–2060. JB JS Open Access. (2023) 8(1). 10.2106/jbjs.Oa.22.00112 36864906 PMC9974080

[3] Tohidi M Brogly SB Lajkosz K Harrison MM Campbell AR VanDenKerkhof E Ten-year risk of complication and mortality after total hip arthroplasty in morbidly obese patients: a population study. Can J Surg. (2019) 62(6):442–9. 10.1503/cjs.017318 31782640 PMC6877380

[4] Woo SH Cha DH Park E-C Kim SJ. The association of under-weight and obesity with mortality after hip arthroplasty. Age Ageing. (2019) 48(1):94–100. 10.1093/ageing/afy161 30304489

[5] Hung CY Chang CH Lin YC Lee SH Chen SY Hsieh PH. Predictors for unfavorable early outcomes in elective total hip arthroplasty: does extreme body mass Index matter? Biomed Res Int. (2019) 2019:4370382. 10.1155/2019/4370382 31687390 PMC6800956

[6] Chen Z Yang G Offer A Zhou M Smith M Peto R Body mass index and mortality in China: a 15-year prospective study of 220 000 men. Int J Epidemiol. (2012) 41(2):472–81. 10.1093/ije/dyr208 22296991

[7] Al Balwi W Al Turki M Memish ZA Fakhoury HMA Al Balwi M Hajeer AH. Age/BMI is a stronger predictor of death in COVID-19 patients than age alone: a pilot study. J Epidemiol Glob Health. (2022) 12(4):548–51. 10.1007/s44197-022-00075-z 36355277 PMC9647239

[8] Fakhoury HMA Yousef Z Tamim H Daher S Attasi AA Al Ajlan A Combined effect of age and body mass index on postoperative mortality and morbidity in laparoscopic cholecystectomy patients. Front Surg. (2023) 10:1243915. 10.3389/fsurg.2023.1243915 38074287 PMC10701421

[9] Evans JT Evans JP Walker RW Blom AW Whitehouse MR Sayers A. How long does a hip replacement last? A systematic review and meta-analysis of case series and national registry reports with more than 15 years of follow-up. Lancet. (2019) 393(10172):647–54. 10.1016/s0140-6736(18)31665-9 30782340 PMC6376618

[10] Ryg J Anru PL Engberg H Jorgensen MG Masud T Christensen K Association of body mass index with all-cause mortality in acutely hospitalized older patients. J Am Med Dir Assoc. (2022) 23(3):507–13.e1. 10.1016/j.jamda.2021.07.015 34389336

[11] Winter JE MacInnis RJ Wattanapenpaiboon N Nowson CA. BMI And all-cause mortality in older adults: a meta-analysis. Am J Clin Nutr. (2014) 99(4):875–90. 10.3945/ajcn.113.068122 24452240

[12] Tjeertes EE Hoeks SS Beks SS Valentijn TT Hoofwijk AA Stolker RJR. Obesity–a risk factor for postoperative complications in general surgery? BMC Anesthesiol. (2015) 15:1–7. 10.1186/1471-2253-15-1 26228844 PMC4520073

[13] Yang TI Chen YH Chiang MH Kuo YJ Chen YP. Inverse relation of body weight with short-term and long-term mortality following hip fracture surgery: a meta-analysis. J Orthop Surg Res. (2022) 17(1):249. 10.1186/s13018-022-03131-3 35473595 PMC9044716

[14] Gurunathan U Anderson C Berry KE Whitehouse SL Crawford RW. Body mass index and in-hospital postoperative complications following primary total hip arthroplasty. Hip Int. (2018) 28(6):613–21. 10.1177/1120700017754058 29734847

[15] Scully W Piuzzi NS Sodhi N Sultan AA George J Khlopas A The effect of body mass index on 30-day complications after total hip arthroplasty. Hip Int. (2020) 30(2):125–34. 10.1177/1120700019826482 30719937

[16] Modig K Erdefelt A Mellner C Cederholm T Talbäck M Hedström M. “Obesity paradox” holds true for patients with hip fracture: a registry-based cohort study. JBJS. (2019) 101(10):888–95. 10.2106/JBJS.18.01249 31094980

[17] Manrique J Chen AF Gomez MM Maltenfort MG Hozack WJ. Surgical site infection and transfusion rates are higher in underweight total knee arthroplasty patients. Arthroplasty Today. (2017) 3(1):57–60. 10.1016/j.artd.2016.03.005 28378008 PMC5365405

[18] Seidell JC Visscher TL. Body weight and weight change and their health implications for the elderly. Eur J Clin Nutr. (2000) 54(Suppl 3):S33–9. 10.1038/sj.ejcn.1601023 11041073

[19] Nilwik R Snijders T Leenders M Groen BB van Kranenburg J Verdijk LB The decline in skeletal muscle mass with aging is mainly attributed to a reduction in type II muscle fiber size. Exp Gerontol. (2013) 48(5):492–8. 10.1016/j.exger.2013.02.012 23425621

[20] Myrskylä M Chang VW. Weight change, initial BMI, and mortality among middle- and older-aged adults. Epidemiology. (2009) 20(6):840–8. 10.1097/EDE.0b013e3181b5f520 19806061 PMC2903861

[21] Guo K Wang X Lu X Yang Y Lu W Wang S Effects of sarcopenia and frailty on postoperative recovery in elderly patients: a prospective cohort study. J Cachexia Sarcopenia Muscle. (2023) 14(6):2642–52. 10.1002/jcsm.13337 37724506 PMC10751444

[22] Gingrich A Volkert D Kiesswetter E Thomanek M Bach S Sieber CC Prevalence and overlap of sarcopenia, frailty, cachexia and malnutrition in older medical inpatients. BMC Geriatr. (2019) 19:1–10. 10.1186/s12877-019-1115-1 31029082 PMC6487020

[23] Lieffers J Bathe O Fassbender K Winget M Baracos V. Sarcopenia is associated with postoperative infection and delayed recovery from colorectal cancer resection surgery. Br J Cancer. (2012) 107(6):931–6. 10.1038/bjc.2012.350 22871883 PMC3464761

[24] Yang Y Sun M Chen W-M Wu S-Y Zhang J. Adverse postoperative outcomes in elderly patients with sarcopenia. BMC Geriatr. (2024) 24(1):561. 10.1186/s12877-024-05066-2 38937671 PMC11212269

[25] Zhang X Deng C Wan Q Zhao R Han L Wang X. Impact of sarcopenia on postoperative pulmonary complications after gastric cancer surgery: a retrospective cohort study. Front Surg. (2023) 9:1013665. 10.3389/fsurg.2022.1013665 36684364 PMC9852346

[26] Dziura J Mendes de Leon C Kasl S DiPietro L. Can physical activity attenuate aging-related weight loss in older people?: the Yale health and aging study, 1982–1994. Am J Epidemiol. (2004) 159(8):759–67. 10.1093/aje/kwh105 15051585

